# ‘It Just Makes Sense to Me’: A qualitative study exploring patient decision‐making and experiences with prostate MRI during active surveillance for prostate cancer

**DOI:** 10.1002/bco2.351

**Published:** 2024-04-02

**Authors:** Ryan Sutherland, Cary P. Gross, Xiaomei Ma, Farah Jeong, Tyler M. Seibert, Matthew R. Cooperberg, William J. Catalona, Shellie D. Ellis, Stacy Loeb, Dena Schulman‐Green, Michael S. Leapman

**Affiliations:** ^1^ Yale School of Medicine New Haven Connecticut USA; ^2^ Yale Cancer Outcomes, Public Policy, and Effectiveness Research Center New Haven Connecticut USA; ^3^ Department of Chronic Disease Epidemiology Yale School of Public Health New Haven Connecticut USA; ^4^ Department of Internal Medicine Yale School of Medicine New Haven Connecticut USA; ^5^ Yale School of Public Health New Haven Connecticut USA; ^6^ Department of Radiation Medicine and Applied Sciences University of California San Diego La Jolla California USA; ^7^ Department of Radiology University of California San Diego La Jolla California USA; ^8^ Department of Bioengineering University of California San Diego La Jolla California USA; ^9^ Department of Urology University of California San Francisco San Francisco California USA; ^10^ Department of Epidemiology and Biostatistics University of California San Francisco San Francisco California USA; ^11^ Department of Urology Northwestern University Feinberg School of Medicine Chicago Illinois USA; ^12^ Department of Population Health Kansas University Medical Center Kansas City Kansas USA; ^13^ Departments of Urology and Population Health New York University Langone Health New York USA; ^14^ Manhattan Veterans Affairs Medical Center New York New York USA; ^15^ New York University Rory Meyers College of Nursing New York New York USA; ^16^ Department of Urology Yale School of Medicine New Haven Connecticut USA

**Keywords:** active surveillance, patient‐centered research, prostate cancer, prostate MRI, qualitative research

## Abstract

**Introduction:**

Although prostate magnetic resonance imaging (MRI) is commonly used in the diagnosis, staging and active surveillance of prostate cancer, little is known about patient perspectives on MRI.

**Methods:**

We performed a qualitative study consisting of in‐depth, semi‐structured interviews of patients with low‐ and intermediate‐risk prostate cancer managed with active surveillance. Interviews focused on experiences with and knowledge of prostate MRI and MRI‐ultrasound fusion biopsy during active surveillance. We purposively sampled patients who received prostate MRI as part of their clinical care, conducted interviews until reaching thematic saturation and performed conventional content analysis to analyse data.

**Results:**

Twenty patients aged 51–79 years (mean = 68 years) participated in the study. At diagnosis, 17 (85%) had a Gleason grade group 1, and three (15%) had a grade group 2 tumour. Overall, participants viewed prostate MRI as a valuable tool that accurately localizes and monitors prostate cancer over time, and they considered prostate MRI central to active surveillance monitoring. We identified five thematic categories related to MRI use: (1) the experiential aspects of undergoing an MRI scan; (2) the experience of visualizing one's own prostate and prostate cancer; (3) adequacy of provider explanations of MRI results; (4) confidence in prostate MRI in decision‐making; and (5) the role of prostate MRI in longitudinal follow‐up, including an interest in using MRI to modify the timing of, or replace, prostate biopsy.

**Conclusion:**

Patients value prostate MRI as a tool that enhances their confidence in the initial diagnosis and monitoring of prostate cancer. This work can inform future studies to optimize patient experience, education and counselling during active surveillance for prostate cancer.

## 1 INTRODUCTION

Despite excellent long‐term cancer outcomes with active surveillance for low‐ and favourable‐intermediate‐risk prostate cancer, patients commonly experience uncertainty when considering initial observational management.^1^ Strategies that improve confidence in the assessments of cancer risk and improve communication may reduce cancer‐related anxiety, enhance patient experience and facilitate the initial selection and adherence to active surveillance.^2^ With excellent local diagnostic accuracy, prostate MRI is seen as a valuable tool for improving the assessments of cancer grade and stage and is now recommended by practice guidelines, including the American Urological Association guidelines, to augment risk stratification for patients managed with active surveillance.^3^ Prostate MRI has been rapidly incorporated into clinical practice in multiple contexts, including prior to prostate biopsy, as a tool for local staging and as a component of active surveillance.^4^, ^5^

Although there is much information available regarding the diagnostic and prognostic accuracy of prostate MRI, little is known about patient experiences with and knowledge of MRI imaging and its role in their treatment decision‐making.^6^, ^7^ Prior observational studies have found that use of prostate MRI is associated with increased use of initial observational management of prostate cancer.^8^, ^9^ Furthermore, previous qualitative studies have found that patient self‐perception of cancer risk and understanding of prognostic reports influence decisions to pursue active surveillance.^10^, ^11^ However, the independent role of MRI in driving selection of or adherence to active surveillance is unknown. Additionally, limited guidance exists on how best to communicate prostate MRI results to patients within the context of prostate cancer care.^12^ Undefined patient experiences and informational needs may contribute to high reported rates of scan‐related anxiety (25%–37%), claustrophobia and feelings of unpreparedness for the test.^13^, ^14^, ^15^ Despite modest performance of MRI to identify early progression during active surveillance, imaging is increasingly used in lieu of biopsy and it is unknown whether or how patients view these tradeoffs.^16^ Therefore, we sought to better understand patient perspectives on the role of prostate MRI during active surveillance for prostate cancer and its impact on decision‐making. The objectives were to characterize patient experiences and preferences regarding prostate MRI as a component of active surveillance and to synthesize recommendations and better align practice with patient needs.^17^

## 2 METHODS

### 2.1 Study design

This was a qualitative study exploring patient experiences related to prostate MRI and tissue‐based gene expression testing during active surveillance. Unique data on patient experiences with tissue‐based genomic testing have been presented separately.^18^ The Yale University institutional review board determined that this study was exempt (45CFR46.101 (b)(2)). We report results in adherence to the Consolidated Criteria for Reporting Qualitative Research.^19^

### 2.2 Patient inclusion criteria and enrolment

At a single tertiary care referral centre in the Northeastern United States, we approached English‐speaking patients with low‐ to favourable‐intermediate‐risk prostate cancer who were enrolled in active surveillance for prostate cancer without planned definitive treatment. Eligible patients had to have received at least one prostate MRI as a component of their clinical management. We identified patients through electronic medical record (EMR) queries and patient referrals from clinicians. To include the perspectives of patients of racial and ethnic backgrounds that are understudied in prostate cancer, we aimed to over‐sample patients who self‐identify as Black and/or Latino to enrich the diversity of the sample.^20^ We approached patients using a combination of strategies including secure EMR messages and phone calls followed by a screening telephone call to assess study eligibility, confirm clinical management with active surveillance and review the study objectives. We informed patients that participation was voluntary, that refusal to participate would not impact their clinical care and that a $50 gift card would be provided as compensation after completed interviews.

### 2.3 Interview procedures and guide

We conducted interviews between January 2021 and December 2021 via Zoom™ (Zoom Video Communications), a secure web‐based video conferencing platform, or by telephone, based on patients' access to technology and preference. At the start of the interviews, the interviewer provided patients with a summary of the study design and objectives and obtained verbal consent. Interviews were conducted by RS, a male researcher with post‐graduate training in qualitative interviewing techniques and study methodology. All interviews were digitally recorded.

We used a semi‐structured interview guide (e.g., open‐ended questions with probes) covering understanding patients' experiences undergoing an MRI, interpreting their prostate MRI results and improving communication between patients and providers. Sample questions included ‘Can you tell me about your understanding of why your doctor recommended that you get a prostate MRI?’; ‘Can you describe what it was like to physically have the MRI scan?’; ‘How do you think that the information about your prostate MRI could have been better explained to you?’; and ‘What is your understanding of what the MRI showed and how did that information shape your healthcare decision‐making process?’ We edited the interview guide reflectively, especially during early interviews, based on participants' understanding of and insights into the research questions.^21^

### 2.4 Data analysis

We deidentified and transcribed all interviews and uploaded them into Dedoose (Version 4.12), a cloud‐based software for qualitative research analysis. We used conventional content analysis to analyse the data, an inductive method to explore phenomena about which little is known.^22^ Two investigators (ML and RS) began by reading transcripts and immersing in the data. They next independently performed line‐by‐line coding by identifying key phrases and emergent concepts in the data. After gaining familiarity with the initial interviews, the research team began creating a preliminary coding framework, using consensus coding to agree on codes, their definitions and the code structure. ML and RS also recorded extensive memos on codes and their discussion, logging patient observations. Lastly, we constructed a matrix to depict thematic categories in relation to prostate MRI use. We shared the final analysis with several study participants to ensure that our interpretations were aligned with their experiences.^23^

## 3 RESULTS

### 3.1 Patient characteristics

A total of 58 patients were identified and screened for enrolment. Twenty‐one patients agreed to participate, were consented and were scheduled to interview with our team. One patient was deemed ineligible after enrolment because of a change in disease risk status and treatment plan, leaving 20 patients who matched our inclusion criteria and completed interviews. Common reasons that participation was declined included no answer to research inquiry (*N* = 30), or stated disinterest in participating in the research study (*N* = 7). We conducted 17 interviews via Zoom and three by telephone. The mean interview time was 48.2 min (range: 33–71 min). The mean age was 68 (range: 51–79) years. Two (10%) patients identified their ethnicity as Hispanic or Latino. Five (25%) identified their race as Black and 15 (75%) as White. At diagnosis, 17 patients (85%) had a Gleason grade group 1, and three patients (15%) had a grade group 2 prostate cancer.

Overall, participants viewed MRI as being important to their diagnosis, treatment decision‐making and clinical management. However, experiences and perceptions of prostate MRI during active surveillance varied. We identified five thematic categories: (1) the experiential aspects of undergoing an MRI scan; (2) the experience of visualizing one's own prostate and prostate cancer; (3) adequacy of provider explanations of MRI results; (4) confidence in prostate MRI in decision‐making; and (5) the role of prostate MRI in longitudinal follow‐up during active surveillance. Table 1 outlines the clinical and demographic characteristics of study participants, and Table 2 provides the semi‐structured interview guide utilized in qualitative interviews. A summary of thematic categories and clinical recommendations based on participant responses is presented in Table 3.

**TABLE 1:** Clinical and demographic characteristics of study participants.

Age (mean, range), years	68 (51–79)
PSA at diagnosis, mean	
Gleason Grade, *n* (%)	
Group 1	17 (85)
Group 2	3 (15)
Ethnicity, *n* (%)	
Hispanic or Latino	2 (10)
Not Hispanic or Latino	18 (90)
Race, *n* (%)	
Black or African American	5 (25)
White	15 (75)
Mean interview length, min	48
Interview medium, *n* (%)	
Zoom videoconference	17 (85)
Telephone	3 (15)

**TABLE 2:** Interview guide for descriptive study of patients undergoing MRI as a component of active surveillance for prostate cancer.

Introduction	We are interested in learning about how new types of scans and tests are used to help patients diagnosed with prostate cancer make decisions about whether they should have their cancer watched (‘active surveillance’) or treated. Prostate MRI and genomic testing are two new forms of testing that are designed to give more information about how aggressive a man's prostate cancer is. As a result, they can help patients make an informed decision about being treated treatment or having active surveillance.
1	Thinking back, what was your experience like in choosing active surveillance rather than some other treatment such as surgery or radiation? (Probe: Who helped you make that decision? What was important to you?)
2	Deciding about active surveillance or treatment for prostate cancer usually involves a lot of discussion about the risks and benefits of each approach. Can you tell me about how information about the risks of your prostate cancer was presented to you? (Probe: Did you find it to be effective? Was there anything that you found to be confusing?)
3	I understand that you had a prostate MRI as part of your evaluation. Can you tell me about your understanding of why your doctor recommended that you get a prostate MRI?
4	What is your understanding of what the MRI showed and how did that information shape your healthcare decision‐making process? (Probe: In what ways did getting the MRI alter, if at all, confidence in your decision? In what ways did MRI alter, if at all, your level of anxiety about the diagnosis, or the decision to move ahead with active surveillance?)
5	How was your overall experience with having the MRI scan? (Probe: How did you feel leading up to it, and at the time? Did you feel prepared? [If anxiety/noise/discomfort: how did you cope with them?] How do you think the experience of the test itself could be improved?)
6	How could the information about your prostate MRI or genomic testing have been better explained to you? (Probe: Does hearing a percentage (for example an X% risk of cancer metastasizing) help you? Or would you prefer to see this information visually in a picture or graph? Would it be more helpful for your doctor to describe risks using words like ‘the risk is high’ or ‘the risk is low’ be better? What remaining questions, if any, do you have about your test results? Are there any unresolved questions?)
7	How would you feel about using an electronic survey to help your doctor understand your preferences about discussing risk estimates? Would you want to complete this before your visit with your doctors or discuss it with them directly?
8	As you know, active surveillance involves close monitoring of prostate cancer. Is having a prostate MRI/genomic testing a component of your monitoring plan? If so, do you think the role of these tests in the monitoring process has been explained in an effective way to you? If not, how could it be improved? Do you think a written or electronic document to track this information over time would be helpful?
9	Is there anything we have not discussed that you think it is important for us to know about how new technologies can help patients with low‐risk prostate cancer with decision‐making?

**TABLE 3:** Summary of thematic categories and clinical challenges identified by patients with potential strategies for practice improvement.

Thematic category	Challenge identified	Data‐informed strategy for practice improvement
Experiential aspects of undergoing prostate MRI	Patients may be unprepared for sensory experiences of prostate MRI	‐ Ordering clinician and/or radiology technician should discuss MRI experience and potential sensory effects (e.g., claustrophobia, noise, anxiety) ‐ Offer written or electronic pre‐procedure instructions for prostate MRI ‐ Adjust existing MRI guidance to include information on pre‐procedure preparations and expectations ‐ Offer anxiolytics or sedation for patients unable or concerned about undergoing MRI ‐ Provide headphones or earplugs to patients experiencing auditory discomfort
Experience of visualizing prostate and prostate cancer	Viewing one's own cancer may induce a range of patient emotions	‐ Urologists and prostate cancer clinicians should ask patients if they would like to view MRI images (e.g., ‘would you like to see the pictures from your prostate MRI?’) ‐ Provide opportunities for collaborative review ‐ Providers should be aware that some patients may wish to not view MRI images
Adequacy of provider explanations of MRI results	Immediate release of MRI report via electronic health record portal	‐ Clinicians and healthcare staff should clarify with patients their ability to access their EMR records to assess computer literacy/health literacy. ‐ Set expectations for timeframe and manner of discussing MRI results (e.g., ‘I will aim to let you know the results of the MRI through MyChart, or would you prefer to have a visit to discuss the results?’) ‐ Centre communication about MRI results to their practical meaning for prostate cancer active surveillance ‐ Creation of patient‐centred MRI reports
Confidence in prostate MRI in decision‐making	Discordance between MRI findings and other clinical parameters may increase uncertainty about active surveillance	‐ Clinicians should inform patients that disagreements between MRI imaging and clinical parameters (false positive or false negative) are possible
Role of MRI in longitudinal follow‐up	Patients may be unfamiliar with limitations of prostate MRI in identifying early Gleason reclassification	‐ Clinicians Communicate potential limitations of prostate MRI in identifying disease reclassification ‐ Healthcare systems and radiologists may provide written summaries to compare MRI findings over time, including potential changes

### 3.2 Experiential aspects of undergoing MRI scan

Patients described physical and emotional experiences when undergoing a prostate MRI. Patients contrasted the experience of a prostate MRI with the invasiveness of a prostate biopsy. Although viewed as non‐invasive, patients conveyed their experiences with claustrophobia or sensory discomfort because of the loud noise of an MRI or felt insufficiently counselled prior to the imaging test:

PT 20: … the noise level that the machine makes while the test is going. It's just totally out of control regardless of the fact that you are given the little plugs to stick in your ear and also a set of headphones, that does nothing whatsoever. I mean, you feel like you're inside a trashcan and someone is beating the trashcan with a stick.

However, other patients expressed a clear preference for prostate MRI as a painless and non‐invasive test, especially when viewed in comparison with prostate biopsy:

PT 15: I feel that MRI is a very, very crucial instrument … And it has been a very efficient, I think, better than the biopsy, which is painful. MRI, even if it is intimidating … it's not painful at all.

A lack of clear guidance informing patients how to prepare for prostate MRI or what to expect during the MRI increased anxiety and confusion for some participants. Furthermore, waiting for the release of MRI reports or uncertainty about what these tests would show heightened concern about imaging findings, and its implications for their prostate cancer.

PT 2: … she was saying to me, ‘Did you do the enema?’ Well, no one said anything to me about doing an enema. But before I went, like an hour before I left the house to go to the MRI appointment, I got on to, I think it was [X] to confirm the appointment. And [she] said, ‘Do the prep first.’ So this caused a little bit of stress.

PT 1: So going into the MRI … there's a little bit of that, ‘Okay, am I going to get the email from MyChart today? Is it going to be the email from MyChart today?’ And when I do, when I'm looking at, what does it tell me? Because obviously, the terms, in there were things that I was, ‘Okay, it says this. What is this?’ I would then Google and look for information and definitions of what this is. ‘What is this an indicator of? What are they trying to say when they say this?’

### 3.3 Experience of visualizing prostate and prostate cancer

By visualizing the prostate, the location and dimensions of suspicious prostate lesions, some patients found MRI to be an informative tool for understanding their prostate cancer diagnosis and recommended clinical management:

PT 2: any information on the structure of what's being seen in the prostate, exactly where the lesion is … I would find helpful.

For some patients, visualizing one's prostate biopsy procedure was reassuring that the cancer was being adequately sampled over time. Patients also commonly connected prostate MRI with biopsy and perceived MRI as being important in refining localization and accuracy of prostate biopsy:

PT 5: It was really interesting and somewhat reassuring that they were really targeting what the issue was … give me as much information as you can.

Some patients found that visualizing one's own prostate imaging during MRI‐ultrasound fusion biopsy enhanced feelings of agency in their prostate cancer care:

PT 10: The MRI … was projected on the screen on a huge 65‐inch TV, and then the biopsy went on while I was fully awake. It was wonderful and fascinating to watch. I'm not the type of person who wants to be in the dark about anything.

Although some patients placed high value on visualizing MRI images, others expressed preferences against viewing the results of their test. Some explained that being confronted with testing images could be anxiety‐provoking because of a lack of sufficient clinical knowledge to interpret results.

PT 13: [I]f I see an MRI picture, and they tell me, it might scare me. The less I see, the less I know, I feel great … too much of a visual is just not a good thing.

### 3.4 Adequacy of provider explanations of MRI results

Some patients expressed a preference for greater information about their prostate MRI findings and their clinical implications. Although some patients felt that discussions about their imaging results were adequate, others expressed interest in greater detail:

PT 19: I was able to watch what was going on and he pointed out areas where he was taking it from. And [he] was very, very thorough in terms of [describing] metastasis, of the potential for that, and the fact that my level of cancer was very low.

PT 8: I would love to have seen the doctor say, ‘Look, here is the picture of the MRI and the size and the location of your first one. And here it is on the second one.’

Some patients were reassured by provider explanations of MRI results placed clearly in the context of their plan for active surveillance monitoring.

PT 1: I was very positive about [active surveillance], because Dr. [X] took the time to explain to me exactly why it was needed and why it was so good … He says, ‘With the MRI, we now use that as a road map to get to certain places that are of concern.’

Tailored conversations, guided by patients' informational needs, were suggested by several patients to improve their understanding of the purpose of diagnostic technologies like MRI. As PT 12 noted, ‘[i]t might be good to ask the patient, “How would you like [this information] presented?” I think there's probably people want percentages, graphs, or they want something in writing, right? Or all of the above. It depends on the patient.’

Several patients noted that receiving MRI imaging reports through electronic health record portals prior to discussion with their clinician increased anxiety and uncertainty:

PT 9: The MRI was bad for me, because of the fact that I got the test, because they posted it to your MyChart online. And so I got the results of the MRI before I was going to talk to the doctor. Usually the doctor’s the guy who tells you the bad news. And of course, results of biopsy, they don't post that, okay? Your doctor talks to you directly about that. But the MRI stated that there were two clinically significant areas of cancer found on the MRI. So unfortunately that was not a good experience.

### 3.5 Confidence in prostate MRI in decision‐making

Patients generally felt that undergoing a prostate MRI enhanced their confidence in selecting and continuing active surveillance by providing greater certainty about the location and risks posed by their prostate cancer. Participants contrasted feelings of certainty with visualizing areas of cancer on an MRI with other tests, such as PSA, which they felt was subject to variation and uncertainty. At the point of initial decision‐making, some patients felt reassured about the ability to localize and track their prostate cancer over time.

PT 15: [My doctor] showed me some of the MRI pictures. He said that in 2018, and this was 2019. So, I could see also. So, I was convinced by those images that it was not actually growing up fast. And it made my decision even better, to see the MRI pictures … So, it was really helpful to know that comparison. To look at the different evolution through the year. MRI I think is a very powerful instrument. Because it's like showing evidence. Showing you, look, there is no growth here. So, why make this decision, which has so many risk, that it may need… So, it gave me more confidence.

PT 8: The PSA testing … I read so many articles and whatnot about how inconsistent it is and how you can't rely on it and how it's not very useful … .MRI … [has] been very important and helpful, and I have a lot of confidence in it, because again, it just makes sense to me.

Some participants were reassured by the perception that they were receiving state‐of‐the‐art imaging evaluations (e.g., 3 T vs. 1.5 T MRI). The perception that higher quality imaging provides more accurate assessments of disease status can increase confidence in selecting active surveillance.

PT 12: Then when I did go to Dr. [X] and we went to the 3T, of course the 3T is much more magnified and it showed something different than the original MRI.

Seemingly discordant findings on prostate MRI compared with prostate biopsy (e.g., PI‐RADS 5 lesion but Gleason grade group 1 biopsy) were a source of uncertainty for some participants. Additionally, although some patients felt MRI could help identify larger, more aggressive cancers or substantial changes in disease status, several reported concerns over sampling randomness, false positives or difficulties in identifying cancerous lesions:

PT 9: The fact that I had the false positives is an aberration of my particular case, but I am still mentally working on personally working on getting to the bottom of why I'm getting the false positives … if you read both MRIs, they're literally almost identical [but the] length of the suspected tumor area is off by one or two tiny increments … And I really wasn't able to satisfy my … answer to why the MRI does call it wrong at times.

### 3.6 Role of prostate MRI in longitudinal follow‐up during active surveillance

Participants generally felt confident in the ability of prostate MRI to identify changes in their disease over time. Clinical evidence of modest performance of MRI in detecting biopsy reclassification was not discussed by participants. The ability to visually identify and track areas of disease over time was consistently seen as reassuring:

ID 15: MRI has been a very efficient way to not only look at the longitudinal evolution of the disease, but also to keep records of the day, of the year, and keep record, and explain visually, what is going on … I feel that MRI is a very, very crucial instrument.

However, some patients expressed difficulty monitoring results over time because of issues accessing prior test results or a desire for a composite document for ease of record keeping. As PT 8 expressed, ‘I would like to have physically seen a side‐by‐side picture of the lesions, and showing me, “You can see here this is this much bigger, and it grew this much over two years. And therefore, if we extrapolate that growth over a two‐year period, we think that you've probably got at least another X years before we're really concerned with it.”’

## 4 DISCUSSION

We examined patient experiences, knowledge and preferences regarding the use of prostate MRI during active surveillance for low‐ and intermediate‐risk prostate cancer. We uncovered distinct thematic categories relating to the experience of undergoing MRI imaging, visualizing one's own prostate, provider communication and the role of MRI in decision‐making and monitoring. Overall, we found that prostate MRI plays a central role in patient perception of cancer risk status and assessments of suitability for initial active surveillance. Participants also expressed confidence in MRI as a reliable and non‐invasive tool to identify disease reclassification. However, we uncovered a range of patient preferences relating to the nature and detail of communication of imaging results. Synthesizing these findings, below we suggest patient‐centred clinical recommendations that can be used for practice improvement.

Our findings provide explanatory information about patient perceptions of prostate MRI imaging during active surveillance selection and monitoring. In this sample, we identified generally favourable patient experiences with prostate MRI. In particular, patients viewed MRI as highly reliable for disease localization and expressed that imaging increased confidence in the decision to select active surveillance and comfort with the ability to identify disease reclassification over time. Therefore, MRI may reduce initial uncertainty about cancer risk, a barrier to active surveillance selection and adherence.^24^ As a result, these findings are aligned with prior studies that have identified associations between prostate MRI use and greater use of active surveillance for prostate cancer.^25^, ^26^

The role of prostate MRI as an alternative to prostate biopsy for active surveillance monitoring was prominent among several participants. Although some felt reassured by using prostate MRI to more accurately direct surveillance prostate biopsies, others viewed imaging as equivalent or superior to biopsy and did not discuss its moderate reliability in identifying progression.^16^ Until more rigorous evidence is available to help understand the role of prostate MRI on long‐term outcomes of active surveillance, clinicians should take note to communicate potential diagnostic limitations of MRI as a stand‐alone modality or replacement for biopsy.

Communication of prostate MRI planning and results shaped the selection, confidence and interpretation of MRI findings. Participants expressed the importance of clarity in delivering test results and centring discussions of prostate MRI results on their practical clinical meaning, including the suitability for continued active surveillance or the need to trigger action such as a repeat prostate biopsy.^27^ Limited communication about pre‐MRI preparations (e.g., need for enema) and the possibility of loud noise or claustrophobia increased anxiety and distress for some patients. Therefore, prostate cancer clinicians should take note to also provide anticipatory guidance for MRI studies beyond what may be provided by radiology centres. Consistent with prior studies in the active surveillance population, we found that patients emphasized the need for information sources to tailor levels of detail to patient preferences and comfort level.^2^

Participants identified new and evolving healthcare system‐level challenges when receiving imaging reports. Delivery of imaging results via electronic health record portals heightened anxiety in some participants, particularly when they were unable to receive explanations by their physicians, similar to findings reported in cancer types.^28^ The Information Blocking Rule of the 21st Century Cures Act, designed to increase accessibility and portability of health data, has resulted in the near immediate release of radiology reports on patient portals. Although these initiatives have increased transparency, our findings highlight potential impacts on patient anxiety and decisional uncertainty, effects that may be pronounced in patients with cancer. To address these concerns, healthcare systems should consider providing clear timelines when results will be discussed as well as increasing telehealth access for more timely counselling. In addition, some participants suggested the need for more concise and comprehensible information accompanying radiologist MRI reports. To improve communication, healthcare systems may also consider integrating simple, plain language summaries of actionable clinical findings and timelines for next steps.^29^

### 4.1 Study limitations

Our data are neither representative of all patients with low‐ and intermediate‐risk prostate cancer nor immediately generalizable across treatment contexts and study populations. We aimed to enrol a diverse sample of patients both clinically and demographically for this study across racial and ethnic groups, and cancer risk strata, and academic versus community affiliation. However, our sampling frame, because of challenges with enrolment during the COVID‐19 pandemic, was restricted to one academic tertiary referral centre. Considering that our study only evaluated the perspectives of patients who selected active surveillance, future studies are needed to evaluate the experiences of patients who discontinued active surveillance or sought early treatment despite having lower risk prostate cancer. As a result of the COVID‐19 pandemic, interviews were conducted remotely over Zoom™ teleconferencing software or via telephone. This requirement excluded patients who lacked access to these technologies and may have affected the interviewer‐interviewee dynamic.^30^

## 5 CONCLUSION

In this qualitative study of patients with low‐ and intermediate‐risk prostate cancer managed with active surveillance, we found that participants held favourable views of prostate MRI and prioritized MRI to improve confidence in cancer risk assessment and its ability to track longitudinal changes over time. Patients shared a range of preferences for information about MRI results and generally emphasized the need for clearer provider discussion of findings and their potential clinical significance.

## AUTHOR CONTRIBUTIONS

Michael S. Leapman, Cary P. Gross, Xiaomei Ma, and Dena Schulman‐Green conceived of the concept and provided oversight for its development. Ryan Sutherland, Michael S. Leapman, and Dena Schulman‐Green developed the interview guides and conducted the analysis. Ryan Sutherland carried out study interviews. All authors provided critical feedback and helped shape the research, analysis, and manuscript.

## CONFLICT OF INTEREST STATEMENT

None.
